# Overexpression of the *Pyrus sinkiangensis LEA4* Gene Enhances the Tolerance of *Broussonetia papyrifera* to the Low Temperature During Overwintering

**DOI:** 10.3390/ijms27020688

**Published:** 2026-01-09

**Authors:** Xiaoxia Bao, Xueying Yang, Xue Wang, Hongliang Xin, Qianqin Li, Saisai Wang, Wenwen Xia, Jin Li

**Affiliations:** College of Life Sciences, Shihezi University, Shihezi 832000, China; v2023bxx@163.com (X.B.); 15299925708@163.com (X.Y.); 17899934396@163.com (X.W.); xhl18899533171@126.com (H.X.); liqianqin314@163.com (Q.L.); wssshzu@163.com (S.W.); xiashzu@126.com (W.X.)

**Keywords:** Korla fragrant pear, *PsLEA4*, wintering, freezing damage

## Abstract

Korla fragrant pear (*Pyrus sinkiangensis*), valued for its unique flavor, suffers from freezing damage in its native Xinjiang. Previous studies indicated a strong correlation between low-temperature stress and the expression of LEA genes, particularly *PsLEA4*. This study cloned *PsLEA4* from *P. sinkiangensis* and overexpressed it in paper mulberry (*Broussonetia papyrifera*). The encoded 368-amino-acid protein is localized to the endoplasmic reticulum. Under −4 °C stress, the proline and soluble protein contents in the overexpressing lines increased to 1.21-fold and 1.36-fold, respectively, compared to the wild type, while relative water content (RWC) reached 1.58-fold. And catalase (CAT), peroxidase (POD), and superoxide dismutase (SOD) activities increased by 9%, 16%, and 38%, respectively. During overwintering, the transgenic line exhibited soluble protein content and RWC at 1.78-fold and 1.49-fold compared to those of the wild type, respectively. Malondialdehyde (MDA) and relative electrolyte leakage (REL) levels were only 66% and 63% of the wild type, while CAT and POD activities reached 1.87-fold, and SOD activity peaked at 2.49-fold. These adaptations were associated with improved cold tolerance and with bud break occurring 7–10 days earlier than in WT the following year. These findings could help to understand the molecular mechanisms of *P. sinkiangensis* for overwintering and provide new genetic resources to breed varieties of pear that can resist cold temperatures.

## 1. Introduction

*Pyrus sinkiangensis*, also known as Korla Fragrant Pear, is a distinctive fruit tree in the Xinjiang region in China. Owing to its excellent comprehensive agronomic traits, this species is widely regarded as a core parent in pear breeding [1]. However, the frequent occurrence of extremely low temperature weather damages the flower buds due to freezing and causes the trunks to crack. This damage can also trigger secondary disasters, such as pear canker, which poses a serious threat to the sustainable development of its industry [2]. Such disasters often cause significant reductions in production and a remarkable decline in fruit quality; it can lead to the destruction of entire orchards and result in sustained economic losses [3]. Therefore, the exploration of key overwintering genes and the cultivation of new germplasms resistant to cold have become the core priority to overcome the bottleneck of damage from freezing and achieve the sustainable development of the *P. sinkiangensis* industry.

Perennial woody plants have evolved complex strategies for their dormancy over the winter to adapt to cold environments [4,5]. This process, which is designated autumn cold acclimation and winter dormancy, is often initiated when the daylight hours shorten and the temperatures drop below a certain threshold [6]. Deciduous species of trees rapidly activate hydrolases under the synergistic regulation of abscisic acid (ABA) and ethylene, which accelerate the shedding of leaves. This process reduces water loss and removes potential pathogens [6,7]. Evergreen species retain their canopy but significantly reduce their rates of photosynthesis during winter [8]. These plants transition to a low-energy dormant state to avoid physiological damage from low temperatures and intense light [9]. Stems and roots are the key dormant organs in perennial deciduous trees. They often enhance the tolerance of buds to cold through mechanisms, such as cellular supercooling and covering the bud surfaces with scales, to ensure safe overwintering [10,11]. At the cellular level, low-temperature signals trigger protective dehydration [12,13]. Water moves from inside the cell to the intercellular space, which effectively prevents mechanical damage to the membrane systems and organelles caused by the intracellular formation of ice crystals [14]. Additionally, plants induce the production of large quantities of antifreeze proteins (AFPs) [15]. These proteins lower the freezing point and prevent ice crystals from recrystallizing. By attaching to the surface of ice crystals, they further inhibit the growth of crystals; this prevents disruption of the cells [16]. To adapt to excessive water loss, starch and other macromolecules within the cells are hydrolyzed into soluble sugars, such as sucrose, trehalose, and raffinose, by the action of enzymes [17,18,19,20]. These sugars not only provide energy as respiratory substrates to keep the plants functioning, but also maintain their osmotic balance and protect the stability of proteins and membrane lipids [21,22,23]. In general, dormancy is one of the core mechanisms used by perennial woody plants to adapt to the cold season [24]. Its multi-level cooperative restructuring at the organ–tissue–cellular levels enables the tree to enter a reversible state of life suspension under extremely cold temperatures to accumulate the energy required for regreening in the spring.

ABA is widely recognized as the core hormone that regulates the dormancy of plants and the stress responses among numerous endogenous signaling molecules [25]. During the acclimation to cold in the fall, the levels of ABA rapidly increase within the plants and activate the expression of downstream signaling pathway genes [25,26]. Studies have shown that ABA acts as a positive regulator of dormancy [27,28]. It promotes the initiation of dormancy, but it also delays the release of dormancy by inhibiting the gibberellin signaling pathway [29]. For example, the levels of ABA in grape (*Vitis vinifera*) buds increase significantly during the early stages of dormancy and then gradually decline to correspond with the release of dormancy [30]. Similar phenomena have also been verified in peach (*Prunus persica*) and cherry (*Prunus avium*) [31,32]. Nine-cis-epoxycarotenoid dioxygenase (NCED), the key rate-limiting enzyme in the biosynthesis of ABA, plays a central role in regulating dormancy. The Arabidopsis double mutant of *AtNCED6* and *AtNCED9* weakened seed dormancy owing to the inhibition of ABA biosynthesis [33]. Conversely, the overexpression of the NCED gene in tomato (*Solanum lycopersicum*) leads to a substantial accumulation of ABA, which thereby delays seed germination and promotes the maintenance of dormancy [34]. Except for regulating dormancy, ABA also mitigates the damage from oxidative stress induced by low temperatures by enhancing the activity of antioxidant enzyme systems, thereby ensuring that the plant survives during overwintering. For example, the activities of SOD, CAT, and POD of three varieties of winter wheat (*Triticum aestivum*) (Dn1, Dn2, and J22) significantly increased during overwintering with decreasing temperatures [35]. ABA also plays a central role in maintaining the balance of cellular water and alleviating the damage to cold stress because it is a key signaling molecule [36]. The overexpression of *TaPYL1-1B* in wheat significantly enhances drought tolerance owing to the increase in sensitivity to ABA [37]. Treatment with ABA can also enhance the relative water content of coffee (*Coffea arabica*) leaves under conditions of limited water [38]. It was demonstrated that ABA signaling can directly or indirectly regulate the expression of hydrophilic proteins, such as the Late Embryogenesis Abundant (LEA)/dehydrins (DHNs). When the content of ABA increases, the activated SnRK2 protein kinase rapidly phosphorylates the ABF/AREB type transcription factors (TFs). These factors then enter the nucleus and recognize and combine with the ABRE *cis*-element on the promoters of the LEA genes, which directly initiates their transcription [39,40]. Under stress, the accumulating LEA proteins preserve cellular water potential and stabilize macromolecular structures via their strong hydrophilicity and chaperone activity. In this way, they constitute a vital defense against cold stress in plants [41].

The LEA protein family is composed of a group of proteins that accumulate in large quantities during late embryogenesis or in the storage tissues. They are extensively involved in the responses of plants to abiotic stresses, such as drought, high salinity, and low temperatures [42,43]. The LEA proteins are typically highly hydrophilic and were originally discovered in cotton (*Gossypium hirsutum*) [44]. Based on their conservative motifs, characteristics of their amino acid sequences, and their phylogenetic relationships, the Pfam database has classified the LEA proteins into eight subgroups, including LEA1 to LEA6, dehydrogenases (DHN), and seed maturation proteins (SMP) [45,46]. To date, the genome-wide identification and analysis of the LEA gene families have been conducted in numerous species of plants whose genomes have been sequenced, including pine (*Pinus tabuliformis*) [47], flax (*Linum usitatissimum*) [48], cucumber (*Cucumis sativus*) [49], cassava (*Manihot esculenta*) [50], and Chinese plum (*Prunus mume*) [51]. Numerous functional studies have demonstrated that the LEA gene significantly enhances the resistance of plants to stress. For example, a genome-wide study in bread wheat (*Triticum aestivum*) identified 179 LEA members, among which *TaLEA4-1* and *TaLEA4-5* were experimentally confirmed to be upregulated under drought stress [52]. A total of 72 LEAs were identified in non-heading Chinese cabbage (*Brassica rapa* subsp. *pekinensis*), and *BcLEA_4-7* and *BrLEA_4-18* were highly sensitive to low temperatures [53]. In tea (*Camellia sinensis*) plants, 11 *CsLEAs* genes responded to low temperatures, high temperatures, drought, and treatment with ABA [54,55]. Overexpressing the wheat *TaLEA* gene in Arabidopsis enhances the tolerance to salt and drought resistance of the transgenic line [56]. The rice (*Oryza sativa*) OsLEA5 protein regulates the expression of antioxidant enzymes by interacting with its TF *ZFP36*, thereby enhancing the resistance to stress during the germination of seeds [57]. The overexpression of wheat *DHN5* in Arabidopsis enhances its tolerance to salt and osmotic stress [58]. These studies provide a crucial foundation to systematically reveal the multifunctional mechanisms of LEA proteins in the responses to stress in plants.

Currently, there are still not many studies on the adaptability of *P. sinkiangensis* to overwintering. Our research team previously used comparative transcriptomics to systematically analyze the dynamic changes in gene expression within the phloem tissue of *P. sinkiangensis* during winter dormancy. The expression of the *PsLEA4* gene significantly increased under low-temperature stress, and its level of expression positively correlated with the fluctuations of temperature in the environment during overwintering (Figure S1). This finding suggests that *PsLEA4* may play a key role in the process of adaptation to low temperatures by *P. sinkiangensis*. *B. papyrifera* is an excellent species of tree that grows rapidly and has been selected and bred by the Institute of Botany, Chinese Academy of Sciences (Beijing, China). However, it is highly sensitive to frost when planted in cold regions, such as northern Xinjiang. This overwintering characteristic is similar to that of *P. sinkiangensis*. Therefore, this study systematically investigated the role of *PsLEA4* during natural overwintering using transgenic *B. papyrifera* as the material, using transgenic and physiological-biochemical analytical methods. This research holds significant implications for enhancing the tolerance of plants to cold and advancing breeding efforts for cold resistance.

## 2. Results

### 2.1. Bioinformatic Analysis of PsLEA4

**Figure 1:**
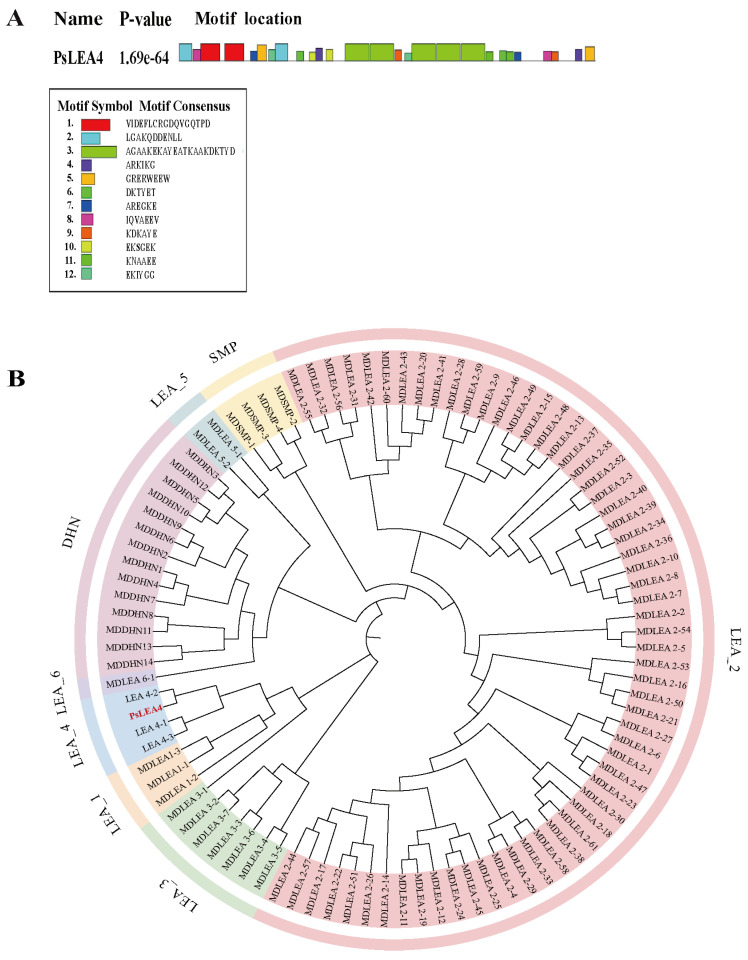
Bioinformatics analysis of *PsLEA4*. (**A**) Predicted conserved motifs of *PsLEA4*. (**B**) Phylogenetic analysis of *PsLEA4*. Apple LEA protein sequences were batch-downloaded from the InterPro database based on their Pfam IDs and used together with *PsLEA4* for the phylogenetic analysis. MEGA 11 software was used to construct an unrooted phylogenetic tree via the Neighbor-Joining method with 1000 bootstrap repetitions to assess the node support. The final phylogenetic tree classified the LEA proteins into eight subfamilies. Each is labeled with a distinct color in the figure. Red, PsLEA4.

The *PsLEA4* gene was cloned from *P. sinkiangensis*. The sequence was 1620 bp long and contained a 1295 bp open reading frame that encoded 368 amino acids. The bioinformatics analysis indicated that the PsLEA4 protein had a molecular weight of 39.67 kDa and a theoretical isoelectric point (pI) of 5.58. This suggests that it carries an overall negative charge. The fat index of this protein was 46.06, and its instability index was 18.59. These values indicate that PsLEA4 is a highly stable hydrophilic protein. The prediction of protein structure indicated that PsLEA4 contains an α-helix as its primary secondary structural element (accounting for 80.43%), and it has low proportions of β-sheets and random coils. Concurrently, this protein exhibits broad intrinsically disordered regions (IDRs), and this feature is consistent with the structure of typical LEA proteins. Additionally, PsLEA4 is predicted to possess both a transmembrane domain and a signal peptide located at positions 28–29, with a probability of 0.3697. This suggests it may exert its biological function through the secretory pathway. The MEME tool identified 12 conserved motifs, including AGAAKEKAYEATKAAKDKTYD, ARKIKG, DKTYET, AREGKE, KDKAYE, EKSGEK, and KNAAEE. These motifs are significantly enriched with hydrophilic and electrically charged amino acid residues, such as Lys (K), Glu (E), Arg (R), and Asp (D). This implies that they could potentially be involved in protection against dehydration, chelating ions, and molecular chaperone functions. Notably, the AGAAKEKAYEATKAAKDKTYD motif exhibits the characteristics typical of acidic transcription activator domains. The tryptophan (W) residue in the GRERWEEW motif functions as a hydrophobic aromatic residue. It often appears at protein–protein interaction interfaces, which suggests that it may mediate specific protein interactions (Figure 1A). We constructed a phylogenetic tree using apple (*Malus domestica*). The view of the LEA proteins as an outgroup further clarifies the evolutionary position of PsLEA4. The phylogenetic analysis revealed that PsLEA4 clustered into a highly supported monophyletic group with known members of the LEA-4 subfamily. Consequently, it was classified into the LEA-4 subfamily and formally designated PsLEA4 (Figure 1B).

### 2.2. Subcellular Localization of PsLEA4

**Figure 2:**
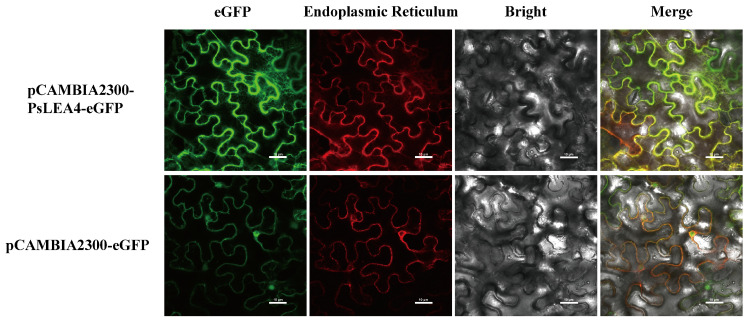
Subcellular localization of the PsLEA4 protein. Bar = 10 μm.

WoLF PSORT 3 and DeepLoc 2.0 predicted that the PsLEA4 protein may be localized to multiple cellular compartments, including the endoplasmic reticulum (ER), cell membrane, extracellular space, and chloroplast. We constructed the fusion expression vector pCAMBIA 2300-PsLEA4-eGFP to determine the actual subcellular localization of PsLEA4 and used the pCAMBIA 2300-eGFP empty vector as a control. Laser confocal microscopy revealed that the control vector pCAMBIA2300-eGFP fluoresced green throughout the entire cell, whereas the fluorescence signal from pCAMBIA2300-PsLEA4-eGFP was specifically localized to the ER. These results confirm that the PsLEA4 protein is indeed localized to the ER (Figure 2)

### 2.3. Phenotypic and Physiological Changes in PsLEA4 Transgenic B. papyrifera Under Low-Temperature Treatment

**Figure 3:**
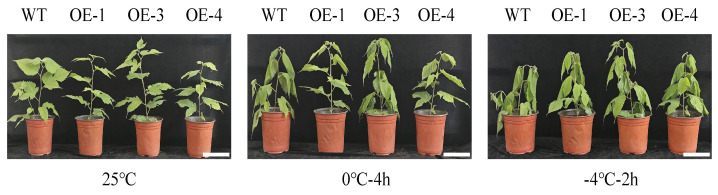
Phenotypic changes in the wild-type and transgenic lines that overexpressed *PsLEA4* in the transgenic *B. papyrifera* under low-temperature stress. Bar = 7 cm.

This study cultivated the transgenic lines (OE-1, OE-3, and OE-4) and WT plants under conditions of 25 °C, 0 °C, and −4 °C to verify the effect of *PsLEA4* on the cold tolerance of the transgenic *B. papyrifera*. No morphological differences were observed between WT and transgenic plants at 25 °C. After 4 h at 0 °C, WT leaves showed slight wilting, whereas OE lines remained turgid or exhibited only mild symptoms. At −4 °C, all genotypes wilted, but damage was consistently less severe in the transgenic lines (Figure 3).

**Figure 4:**
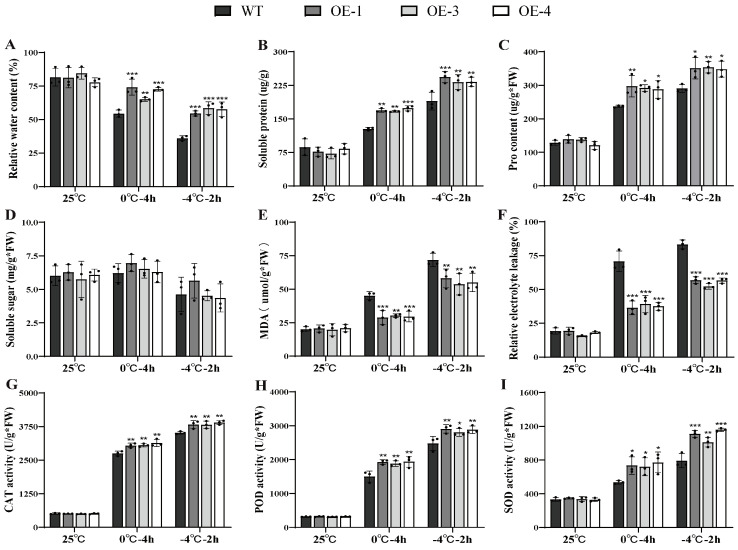
Physiological changes in the wild-type and *PsLEA4* that overexpressed *B. papyrifera* under low-temperature stress. (**A**) RWC (%). (**B**) Soluble protein. (**C**) Pro content. (**D**) Soluble sugar. (**E**) MDA content. (**F**) REL (%). (**G**) CAT activity. (**H**) POD activity. (**I**) SOD activity. Data are means ± SD of three replicates. Asterisk(s) indicate significant difference between the wild-type and transgenic plants: ns (*p* > 0.05), * *p* < 0.05, ** *p* < 0.01, and *** *p* < 0.001.

Under non-stressed conditions, the three independent over-expression lines showed physiological indices similar to those of WT plants. After cold treatment, however, the OE lines exhibited significantly better physiological performance than WT (Figure 4). RWC in the OE lines consistently remained higher than in the WT lines at both 0 °C and −4 °C, peaking at 1.58 times the WT level (Figure 4A). This suggests that *PsLEA4* expression is associated with enhanced cellular water retention. Under low-temperature stress, we observed a significant increase in osmotic regulatory substances such as soluble proteins. At 0 °C and −4 °C, the soluble protein accumulation in the OE strains was 1.36 times that of the WT (Figure 4B). The proline content in the OE lines was higher than that in the WT at both 0 °C and −4 °C (Figure 4C). Notably, although the content of soluble sugar varied under the low-temperature conditions, no significant differences were observed between the transgenic lines and the WT lines (Figure 4D). This suggests that the observed increase in proline and soluble-protein accumulation may improve osmotic adjustment and thereby help maintain cellular water retention, potentially alleviating metabolic imbalances caused by excessive water loss and internal-environment disruption under low-temperature stress. MDA is a byproduct of the lipid peroxidation induced by reactive oxygen species (ROS), and with the REL value, they reflect the extent of damage to the plasma membrane [59]. At 0 °C and −4 °C, WT leaves accumulated 1.4-fold more MDA and showed higher REL than all OE lines, indicating reduced membrane peroxidation and leakage in the transgenics (Figure 4E,F). These data suggest that the presence of *PsLEA4* may contribute to cold tolerance by helping to maintain membrane stability.

The antioxidant enzyme system is used as a key defense mechanism for plants to accumulate ROS when responding to abiotic stress. They maintain their redox balance by scavenging excess ROS [60]. At 0 °C and −4 °C, CAT activity in OE lines consistently surpassed WT, reaching a maximum of 1.12-fold (Figure 4G). POD followed suit, rising above WT at both temperatures and peaking at −4 °C (Figure 4H). SOD maintained a 1.38-fold advantage over WT at −4 °C (Figure 4I).

In summary, the presence of *PsLEA4* was associated with higher cellular water retention, improved osmotic adjustment, reduced membrane injury, and elevated antioxidant protection in transgenic *B. papyrifera* under cold stress.

### 2.4. Phenotypic and Physiological Changes in the PsLEA4 Transgenic B. papyrifera During Natural Overwintering

**Figure 5:**
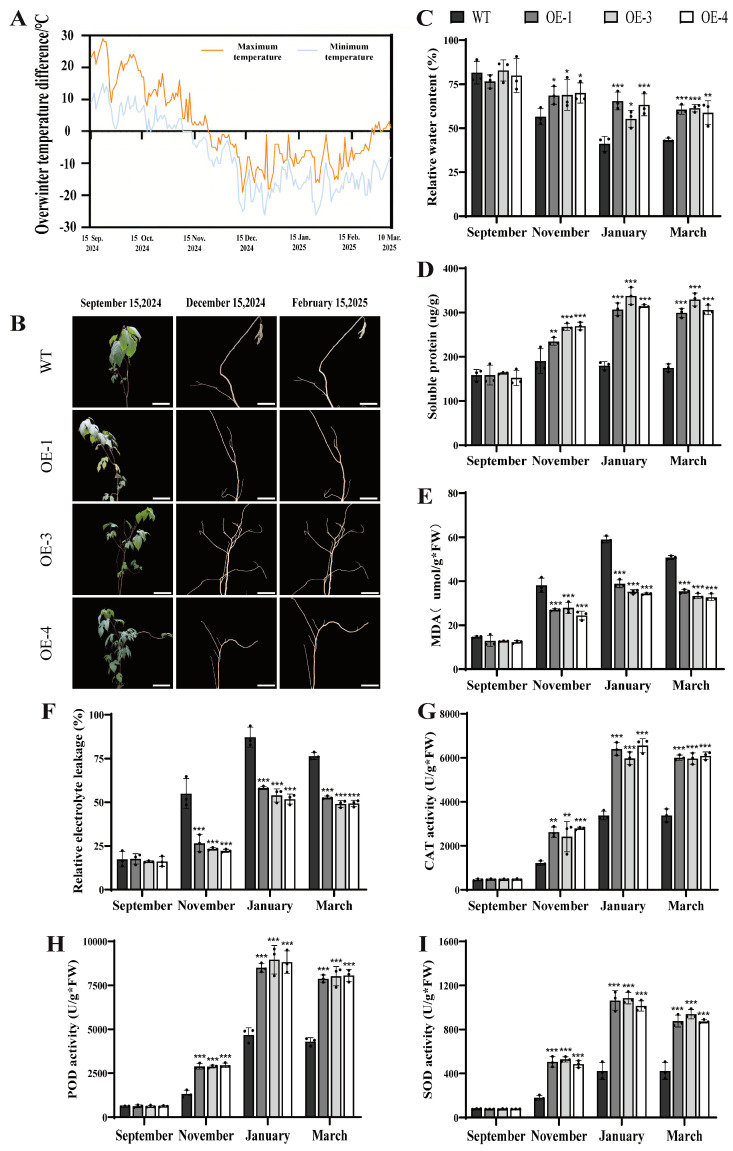
Phenotypic and physiological changes in *B. papyrifera* transgenic for *PsLEA4* during natural overwintering. (**A**) Daily Extreme Temperature Conditions at Sampling Sites During the Wintering Period. (**B**) Phenotype observation of *B. papyrifera* under natural overwintering environment. (Bar = 7 cm). (**C**) RWC (%). (**D**) Soluble protein. (**E**) MDA content. (**F**) REL (%). (**G**) CAT activity. (**H**) POD activity. (**I**) SOD activity. Data are means ± SD of three replicates. Asterisk(s) indicate significant difference between the wild-type and transgenic plants: ns (*p* > 0.05), * *p* < 0.05, ** *p* < 0.01, and *** *p* < 0.001.

This study evaluated the impact of the *PsLEA4* gene on the overwintering capacity of the transgenic *B. papyrifera* by conducting a 7-month field trial to observe the phenotypes of the WT and transgenic lines that overwintered from September 2024 to March 2025 at the Xinjiang trial site. Concurrently, we recorded the daily variations in extreme temperature at the sampling points. The temperatures at the sampling site gradually decreased starting in September and remained consistently low at approximately −25 °C from December to February of the following year, and then began to increase (Figure 5A). Both WT and OE plants senesced and shed all leaves in November, with no genotype difference in defoliation time or branch architecture (Figure 5B). These observations suggest that any improvement in overwintering performance associated with *PsLEA4* may stem from enhanced intrinsic physiological resistance rather than from changes in visible morphological traits.

In September, the WT and OE lines’ physiology did not differ. After temperatures fell in November, the OE lines maintained higher RWC, osmolytes, and antioxidant capacity than WT (Figure 5). During the period from November to March of the following year, the RWC of the OE lines remained consistently higher than that of the WT (Figure 5C), indicating that the presence of *PsLEA4* is associated with reduced winter desiccation. Moreover, the content of soluble protein in the three over-expression lines began to accumulate starting in November. It exceeded 300 µg/g by January, while the WT remained below 200 µg/g. This state continued through March (Figure 5D). The positive correlation between soluble protein dynamics and RWC suggests that the presence of *PsLEA4* may contribute to cellular water retention, possibly through enhanced accumulation of osmotic regulators. The content of MDA revealed that the OE lines exhibited lower levels of peroxidation of the membrane lipids than the WT during the overwintering period. Notably, the levels of MDA in the WT in January were 1.66-fold higher than those of the OE lines (Figure 5E). The REL results were highly consistent, and the WT reached a peak of 87% in January. Moreover, the REL values were higher than those of the OE lines throughout the low-temperature period (Figure 5F). The concomitant decreases in MDA content and REL suggest that the presence of *PsLEA4* may contribute to membrane stability, presumably by alleviating oxidative damage and helping to preserve membrane structural integrity.

This study examined the activities of CAT, SOD, and POD to clear the ROS in the transgenic *PsLEA4* lines. The activities of these three enzymes were upregulated in both the WT and transgenic lines during the overwintering period. This study examined the activities of CAT, SOD, and POD to clear the ROS in the transgenic *PsLEA4* lines. The activities of these three enzymes were upregulated in both the WT and transgenic lines during the overwintering period. From November to March, the combined activities of CAT, POD, and SOD in the OE lines stayed significantly above WT levels. In the coldest month (January), these enzymes were 1.87-fold, 1.87-fold, and 2.49-fold higher in OE than in WT, respectively, and the advantage persisted until overwintering ended (Figure 5G–I). The coordinated up-regulation of antioxidant enzymes was associated with improved oxidative stress tolerance in OE plants. This was accompanied by lower MDA content compared with WT, suggesting that the presence of *PsLEA4* may help maintain membrane stability, presumably through reduced oxidative damage.

In summary, visible overwintering phenotype remained unchanged in PsLEA4-overexpressing *B. papyrifera*. However, transgenic plants exhibited coordinated increases in cellular water retention, osmotic adjustment, membrane stability, and antioxidant defense, physiological changes that may collectively contribute to their improved internal cold tolerance.

### 2.5. Growth Changes in Transgenic PsLEA4 B. papyrifera Before and After Overwintering and Post-Winter Sprouting Status

**Figure 6:**
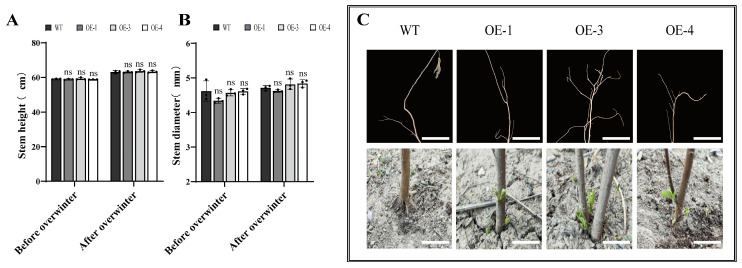
Analysis of the growth indicators during natural overwintering and the status of root sprouting after overwintering in *B. papyrifera* that overexpressed *PsLEA4*. (**A**) Plant height before and after overwintering. (**B**) Stem diameter before and after overwintering. (**C**) Status of sprouting roots after overwintering. Bar = 7 cm. Asterisk(s) indicate significant difference between the wild-type and transgenic plants: ns (*p* > 0.05).

This study evaluated the impact of *PsLEA4* on the winter survival and capacity of *B. papyrifera* to recover its growth by comparing the changes in plant height and stem diameter between the WT and transgenic lines before and after overwintering. There were no significant differences in the height or stem diameter between the WT and OE plants at these two times (Figure 6A,B). This indicated that the expression of *PsLEA4* does not affect the morphological maintenance during dormancy. We subsequently observed that the roots sprouted in the plants on 12 April 2025. All the OE lines had begun budding, while the WT plants exhibited no signs of sprouting at this stage (Figure 6C). The transgenic lines advanced sprouting by 7–10 days compared to the WT. This phenomenon indicates that *PsLEA4* not only enhances the overwintering adaptability of *B. papyrifera* but also significantly promotes its post-winter recovery growth process.

## 3. Discussion

The tolerance of plants to cold is a multi-level and highly coordinated physiological process with a series of response mechanisms, including the maintenance of membrane system stability, accumulation of osmotic regulatory substances, activation of antioxidant defenses, and biosynthesis of antifreeze proteins. These responses are precisely coupled in both time and space, and together, they form the adaptive strategy of plants in response to low-temperature stress [61,62]. In the present study, PsLEA4-overexpressing *B. papyrifera* concurrently exhibited favorable adjustments in cellular water status, osmotic regulation, membrane stability, and antioxidant capacity under low temperature. These correlations suggest that *PsLEA4* may contribute to the cold-adaptive strategy of this species, although causality awaits knockout or silencing validation. To more clearly articulate the findings of this study and their significance, the following analysis will examine aspects including water balance, regulation of osmotic pressure and nitrogen reserves, and maintenance of redox homeostasis. The study’s limitations and future directions will also be summarized.

### 3.1. Association Between PsLEA4 Expression and Improved Water Retention Under Frost Stress

The most direct response of plants under low-temperature stress is an imbalance of water [63,64]. In this study, the relative water content of the transgenic lines remained consistently higher than that of the WT during both artificial low-temperature stress and natural overwintering. This indicates that *PsLEA4* significantly enhances the ability of plants to retain water. This result is consistent with the functions of LEA genes in Arabidopsis and potato (*Solanum tuberosum*) [65,66]. The high hydrophilicity and structural stability of the LEA proteins are considered the key molecular basis used to keep the cells hydrated [67,68]. It has been demonstrated that these proteins can cooperate to respond to dehydration stress by replacing water and functioning as molecular chaperones [69]. We hypothesize that *PsLEA4* may establish a stable hydration environment in the cells by a similar mechanism, thereby alleviating the cellular dehydration induced by the cold. As the winter temperatures grow colder, plants often use active or passive dehydration strategies to prevent the accumulation of intracellular ice crystals on organelles and membrane systems that can cause mechanical damage. However, excessive water loss can also lead to serious tissue dehydration and even death [6]. Plants balance this risk by evolving multiple adaptive mechanisms, including the accumulation of osmotic regulators and enhancement of the activities of antioxidant enzymes [70,71]. These strategies reduce the loss of water and maintain the stability of the cellular structures. They also lay the foundation to resume normal physiological activities in the spring. Notably, the transgenic line maintained higher RWC yet exhibited significantly less cold injury than the WT. These observations suggest that *PsLEA4* may contribute to balancing water retention and limiting freezing damage, although its potential influence on ice-crystal formation or endogenous antifreeze compounds awaits direct examination.

### 3.2. PsLEA4-Overexpressing Lines Show Elevated Osmolyte and Nitrogen Reserves Associated with Cellular Stability

The accumulation of osmotic regulatory substances may be another key mechanism to enhance the cold tolerance of transgenic *B. papyrifera* mediated by *PsLEA4*. The transgenic plants initiated a significant accumulation of soluble proteins from the early stages of overwintering and continued this until the end of the overwintering period. This dynamic process not only enhances the ability to conduct osmoregulation but also stabilizes the cell membranes and protein structures after water exocytosis. This finding is consistent with our observations in seedling-stage experiments and with previous research on Arabidopsis, Satsuma mandarin (*Citrus unshiu*), and foxtail millet (*Setaria italica*) [72,73,74]. Except for maintaining the osmotic equilibrium, the accumulation of soluble proteins may also be used as a reserve of nitrogen (N), thereby providing an immediate source of N and functional proteins for spring growth [75]. This mechanism is consistent with the findings of N fixation studies on alfalfa [76]. In this study, the transgenic lines sprouted early in the spring and probably benefited the plant in part from the protein reserve mechanism promoted by *PsLEA4*. This enabled the plants to rapidly initiate cell repair and growth, which reduced their dependence on the assimilation of N from the spring soils. This strategy is similar to the mechanisms of N reserve in poplar (*Populus* sp.) trees during winter dormancy [77]. Based on this, it is proposed that *PsLEA4* functions through a double pathway. On one hand, as a highly stable hydrophilic protein, its own substantial accumulation directly expands the osmotic protection substance pool. On the other hand, its molecular chaperone activity may directly recognize and stabilize various endogenous functional proteins prone to inactivation under low temperatures, thereby indirectly maintaining cellular protein homeostasis and synthetic capacity, and promoting the establishment of a protective “protein reserve pool.” This reserve provides osmotic support during winter and can be rapidly mobilized in spring to supply nitrogen for recovery and growth.

Proline is also a widely studied osmotic regulator whose intracellular accumulation significantly enhances plant cold tolerance. Part of its mechanism involves inhibiting the nucleation of intracellular ice crystals [78]. At the molecular level, low-temperature stress triggers an increase in cytoplasmic Ca^2+^ concentration, which in turn promotes the nuclear translocation of the phosphorylated Inducer of C-repeat binding factor (CBF) Expression 1 (ICE1) protein [79]. As a transcription activator, ICE1 induces the expression of transcription factor genes such as CBF1/2/3 [80]. Subsequently, CBF proteins bind to C-repeat/dehydration-responsive element (CRT/DRE) cis-acting elements within the promoters of key proline synthesis enzyme genes, for example, Δ1-pyrroline-5-carboxylate synthetase 1 (P5CS1) and Δ1-pyrroline-5-carboxylate reductase (P5CR), directly upregulating their transcriptional levels [81]. Concurrently, the cold signal blocks proline degradation pathways by inhibiting proline dehydrogenase activity [82]. Additionally, the ABA signaling pathway is activated, with its core kinase sucrose non-fermenting 1-related protein kinase 2 (SnRK2) capable of phosphorylating and activating the P5CS enzyme [83]. These synergistic regulatory mechanisms substantially enhance the synthetic pathway flux from glutamate to proline, enabling massive proline accumulation within hours. This ultimately boosts cold tolerance by increasing cellular osmotic pressure. On this basis, In summary, this study proposes that *PsLEA4* may function as a key regulator in the proline synthesis pathway. Through its molecular chaperone activity, it directly stabilizes and activates the rate-limiting enzyme P5CS, thereby robustly ensuring the highly efficient operation of the proline synthesis pathway at the post-translational level. If validated, this mechanism would reveal the functional evolution of LEA proteins from passive protectors to active metabolic regulators, providing a novel integration point for the molecular network governing plant cold tolerance.

In summary, this study proposes that *PsLEA4* protects the cell membrane system by converging soluble proteins and the proline synthesis pathway. One aspect involves the explosive accumulation of proline rapidly elevating cytoplasmic osmotic pressure, effectively counteracting osmotic dehydration caused by extracellular ice formation and alleviating mechanical stress on the membrane system. Simultaneously, proline inhibits intracellular ice crystal formation, preventing direct penetration of membrane structures by ice crystals. In the other aspect, accumulated soluble proteins, including *PsLEA4*, jointly form a stable hydration protective layer with proline at the biomolecular and membrane interfaces. This layer directly maintains membrane lipid fluidity and the functional conformation of membrane proteins, thereby systematically enhancing the structural integrity of cells in frozen–dehydrated environments.

### 3.3. Association Between PsLEA4 Expression and Maintained Redox Balance via Membrane Protection and Antioxidant Enhancement

During the natural overwintering, the activities of CAT, POD, and SOD in the transgenic lines remained consistently higher than those in the WT. Moreover, the content of MDA was maintained at consistently low levels. In addition, this result aligns with the trend under artificial low-temperature stress during the seedling stage. It indicates that *PsLEA4* may effectively alleviate oxidative damage by precisely regulating the metabolism of ROS. This is also consistent with the mechanism of rice *OsLEA5* enhancing cold tolerance by regulating the genes for antioxidant enzymes [84]. This advantage of systemic antioxidants, which persists from the early stages of stress to the end of overwintering, indicates that its function is far more than a transient stress response. It prefers to establish a continuous physiological protective state, and this protective effect may be closely related to the structural characteristics of the PsLEA4 protein. This protein interacts with biological membranes by a molecular shield mechanism because of its abundance of α-helices and IDRs, which prevents the membrane phase transitions induced by cold and subsequent structural collapse [85]. Similar functions were verified in studies of the overexpression of *SiLEA5* in tomato [86], and they are also consistent with the behavior of Arabidopsis *LEA7* at protecting members under dehydration conditions and the molecular shield hypothesis proposed by the Tunnacliffe team [87,88]. The alleviation of membrane damage may originate from a two-term cooperative mechanism. On the one hand, *PsLEA4* directly combines with and stabilizes the lipid structures of membranes, which alleviates the membrane phase transitions induced by cold and the ion leakage on a physical level, thereby reducing the original triggers for the production of ROS. Alternatively, the sustained elevation of the activities of antioxidant enzymes, such as CAT, POD, and SOD, in the transgenic lines significantly increased their capacity to clear existing ROS. The high activity of antioxidant enzymes and low content of MDA together form a self-reinforcing positive protective cycle. This mechanism is similar to the reported function of the Arabidopsis dehydrogenase gene *AtRab18* [89].

Studies have shown that ABA signaling can directly or indirectly regulate the expression of hydrophilic proteins, such as the LEAs and DHNs [90,91]. Moreover, the LEA proteins can mitigate oxidative damage by regulating the activity of antioxidant enzymes and maintaining the balance of ions, and interact with other proteins in the ABA signaling pathway to enhance the adaptation of plants to abiotic stress. This study verified this mechanism during the natural overwintering period of woody plants, thus highlighting the crucial role of the LEA proteins in maintaining the long-term balance of oxidation–reduction.

### 3.4. Summary, Limitations, and Future Prospects

Field observations revealed that *PsLEA4* overexpression did not alter the overall morphology of paper mulberry trees, yet it consistently correlated with enhanced water retention capacity, improved osmotic regulation ability, and high antioxidant capacity. These correlated traits appeared to reduce damage and increase metabolic reserves, and matched the earlier spring bud burst observed in transgenic lines. Compared with studies that report only expression patterns, our stable transformation plus field tracking provides a testable candidate for tree breeding.

The primary limitation of this study is that the evidence remains at the correlational level. Specifically, due to the unknown number of transgenic copies, it is impossible to conduct precise analyses of how gene dosage effects influence phenotypes. Furthermore, while our whole-plant approach identified valuable correlations, deciphering tissue-specific responses requires more refined experiments using mature trees with clearly defined genetic backgrounds.

Future research should, therefore, focus on establishing causality and elucidating the mechanism. The immediate steps include generating single-copy transgenic lines to quantify the dosage-phenotype relationship and employing CRISPR/Cas9-mediated knockout or silencing to confirm the gene’s necessity for the observed traits. Concurrently, foundational work is needed to assemble a chromosome-level genome for *B. papyrifera* to enable the systematic characterization of its native LEA/DHN family, which is currently poorly annotated. Mechanistically, once dosage is controlled, integrating ABA-pathway mutants with multi-omics approaches will help determine whether *PsLEA4* functions downstream of or in parallel to established cold-response pathways like the CBF regulon. Ultimately, integrating a functionally validated *PsLEA4* module with a defined copy number into elite germplasm could transform it from a correlation-based candidate into a precise tool for developing climate-resilient forestry varieties.

## 4. Materials and Methods

### 4.1. Plant Materials

In this study, the hybrid *B. papyrifera* was cultivated in an experimental field located in the Shihezi region (84°89′ N, 44°30′ E). This site is situated in the central part of Northern Xinjiang, China, and is characterized by a topography that slopes from southeast to northwest, with mountainous areas, deserts, and plains as the primary landforms. Shihezi exhibits a typical temperate continental arid climate characterized by scarce annual precipitation, dry conditions, and approximately 2300–2700 h of sunshine a year. The soils of this region predominantly consist of gray desert soils and brown calcareous soils, which are common in irrigated agricultural zones. The experimental site was converted from former orchard land, and it had a history of a foundation of agricultural cultivation practices that provided favorable field management conditions for the cultivation of *B. papyrifera*. *P. sinkiangensis* was cultivated by the pear variety resource preservation garden of the Institute of Agricultural Science, Korla, Xinjiang Uygur Autonomous Region, which is located at 41°49′34.621″ N, 86°12′2.317″ E.

### 4.2. Cloning of the PsLEA4 Gene and Construction of the Plant Expression Vector

Total RNA was isolated from the phloem tissue of *P. sinkiangensis* using the RNAisoPlus Kit according to the manufacturer’s instructions (TianGen Biotech, Beijing, China). The cDNA was synthesized using a reverse transcription kit (TRAN, Beijing, China). Specific primers for the *PsLEA4* gene were designed using Primer 5.0 software (Table S1). Amplification of the cDNA using reverse-transcription PCR (RT-PCR, Thermo Fisher Scientific, Waltham, MA, USA) yielded a fragment that encoded *PsLEA4*. The PCR product was identified by 0.1% agarose gel electrophoresis, purified, and cloned into the pMD19T vector. *Escherichia coli* DH5α was transformed, and the positive colonies were screened and then verified by DNA sequencing. Finally, the correct amplified segment was ligated into the KpnI and BamHI sites of the plant expression vector pCAMBIA 2300. The resulting plasmid was then transformed into *Agrobacterium tumefaciens* GV3101 using quick-freezing with liquid nitrogen. Positive colonies were selected and amplified for subsequent experiments.

The subcellular localization of the PsLEA4 protein was determined by first designing specific homologous recombination primers using SnapGene 8.0.0 software. BamHI and PstI were selected as the restriction sites (Table S1). The full-length sequence of the *PsLEA4* gene with the stop codon deletion was amplified by RT-PCR. Subsequently, the amplified product was ligated into the plant expression vector pCAMBIA 2300-eGFP, which had been cut by both BamHI and PstI to construct the pCAMBIA 2300-PsLEA4-eGFP fusion expression vector. In this vector, the N-terminus of the green fluorescent protein (GFP) was placed under the control of the CaMV 35S promoter.

The constructed pCAMBIA 2300-eGFP and pCAMBIA 2300-PsLEA4-eGFP recombinant plasmids were transformed into competent *A. tumefaciens* GV3101 cells using liquid nitrogen rapid freezing. The colonies were identified as positive by PCR and sequencing, grown to a larger volume, and saved for future use.

### 4.3. Transformation of the Hybrid B. papyrifera and the Acquisition of Transgenic Positive Lines

Transgenic plants that overexpressed *PsLEA4* were obtained by genetically transforming the wild-type (WT) hybrid paper mulberry leaves using *A. tumefaciens* GV3101. The leaves were cut into 1 cm^2^ pieces under aseptic conditions and precultured in the dark on MS medium for 36 h. Activated *A. tumefaciens* GV3101 that harbored the target gene was resuspended in liquid MS medium. The leaves were immersed for 15–20 min, and the excess bacterial solution was blotted off with sterile filter paper. The leaves were then transferred to MS media for co-cultivation in the dark for 48 h. The explants were then transferred to selection medium that contained antibiotics and the compounds 6-benzylaminopuridine (6-BA) and naphthaleneacetic acid (NAA) (MS + 2.0 mg/L 6-BA + 0.5 mg/L NAA) to induce the shoots that were resistant to the antibiotics. After 60 days, the induced shoots were excised and transferred to rooting media (1/2 MS + 0.5 mg/L indole-3-acetic acid [IAA]) for culture (Figure S2). All the media and reagents were purchased from Sangon Biotech (Shanghai) Co., Ltd. (Shanghai, China).

Subsequent screening was used to select regenerated seedlings that were 3–5 cm high and had multiple branches and well-developed root systems. These seedlings were acclimatized in a tightly sealed bottle under strong light for 15 days before they were transplanted into a mixed substrate peat soil: vermiculite: perlite (3:1:2, *v*/*v*/*v*) and cultivated in a growth chamber (25–28 °C, relative humidity [RH] 65–70%, light intensity 7000 lx, photoperiod 16 h light/8 h dark). Paper cups were used to cover the pots, so they retained moisture during the initial period of transplantation with regular misting. After 40 days, the surviving plants were selected for subsequent experiments.

The genomic DNA was extracted from the plants using a DNA extraction kit (TianGen Biotech). The positive transgenic lines were identified by PCR. The WT plant DNA served as the negative control, and the recombinant plasmid served as the positive control, to detect the presence of the target transgenic sequence. Subsequently, mRNA expression levels were detected by quantitative real-time PCR (qRT-PCR) (Table S1 and Figure S3). A total of 120 explants were transformed in this experiment. Among them, 57 passed kanamycin screening, and 47 tested positive by PCR, yielding a positive rate of 39.16%. From these, OE-1, OE-3, and OE-4 were selected as representative lines. Not only did they exhibit the highest *PsLEA4* expression levels, but their growth rates were also fully consistent with robust wild-type controls, thereby eliminating developmental biases in subsequent stress experiments.

### 4.4. Instant Transformation of the Tobacco Leaves

Instant transgenic tobacco leaves were obtained by selecting healthy *Nicotiana benthamiana* plants, which were cultivated for 4–5 weeks under conditions of 25–28 °C, 65–70% RH, 7000 lx light intensity, and a 16 h light/8 h dark cycle. The culture of *A. tumefaciens* that contained the target gene was activated, and the bacterial pellet was collected by centrifugation at 4000 rpm for 5 min and resuspended in a solution that contained 200 mmol/L MES (2-morpholinoethanesulphonic acid), 20 mmol/L magnesium chloride (MgCl_2_), and 200 mmol/L Acetosyringone (AS). Subsequently, the bacterial suspension was injected into the back of tobacco leaves using a disposable syringe with the needle removed. After injection, the tobacco leaves were cultured in the dark for 24 h and then cultivated in the light for 1–2 days. The leaves from the injection site were observed under a confocal scanning microscope (C2; Nikon Instruments Corporation, Tokyo, Japan) to examine the subcellular localization of the gene.

### 4.5. Experimental Treatment

#### 4.5.1. Laboratory Short-Term Low-Temperature Treatment

This study analyzed the effects of low-temperature stress using 60-day-old, uniformly growing wild-type *B. papyrifera* and three independent *PsLEA4* transgenic homozygous lines (OE-1, OE-3, OE-4) as materials. Each genotype constituted one treatment group, with each group containing three biological replications (n = 3). Each replication was developed from a single independent tissue-cultured seedling.

First, leaves in similar states were collected under standard culture conditions at 25 °C. They were quick-frozen in liquid nitrogen and stored at –80 °C as the room-temperature control (0 h). Subsequently, the remaining plants were transferred to a JC-200CL low-temperature incubator (Qingdao Jingcheng Instrument Co., Ltd., Qingdao, China). They were first treated at 0 °C under dark conditions for 4 h. The incubator was then uniformly cooled at a rate of ≤ 1 °C min^−1^ while maintaining darkness and without opening the door. After reaching −4 °C, the cooling process was timed for an additional 2 h. At the conclusion of each temperature stage, leaves exhibiting similar states were rapidly collected using pre-chilled forceps at the corresponding set temperature (0 °C or −4 °C). These leaves were flash-frozen in liquid nitrogen and stored at −80 °C. The entire sampling process avoided any thawing or warming steps, ensuring all measured data represented immediate responses to low-temperature stress.

#### 4.5.2. Field Natural Overwintering Treatment

To evaluate the natural overwintering performance of transgenic plants in detail, the wild-type and transgenic plants subjected to the aforementioned low-temperature treatment, along with the remaining transgenic lines, were transplanted to the experimental field (44°30′ N, 84°89′ E) on 20 May 2024. Field sampling was conducted on 15 September 2024; 15 November 2024; 15 January 2025; and 10 March 2025.

Before sampling, a portable thermometer was used to confirm the surface temperature of the branches (≤0 °C). The phloem was immediately peeled back the phloem using a pre-chilled blade and placed in liquid nitrogen for rapid freezing (to prevent frost damage from water loss through the wound, immediately wrap the cut surface with Parafilm sealing tape). Upon return to the laboratory, samples were stored at −80 °C until analysis. Throughout the process, samples underwent no warming or thawing steps; measured data reflect immediate physiological levels under natural overwintering conditions.

#### 4.5.3. Explanation for Failure to Obtain Phloem During Laboratory Stage

The basal diameter of 60-day-old tissue-cultured seedlings was 1.2 ± 0.1 mm, with no detachable lignified bark yet formed. Preliminary tests indicated that the fresh weight of individual phloem tissue was less than 2 mg, below the minimum sample requirement (30 mg) for enzymatic assays (SOD, POD, soluble sugars, proline). Therefore, only leaves were collected during the laboratory phase; phloem-related parameters will be measured during the field overwintering stage.

#### 4.5.4. Sampling Strategy

For laboratory stress experiments, leaves were collected from each independent biological replicate plant. To obtain a representative value for that plant, multiple collected leaves were pooled to form a technical sample for analysis. The sampling strategy for field natural overwintering experiments is consistent with this approach.

### 4.6. Determination of the Physiological Indicators

The RWC of the leaves was determined by weighing. There were three replicates per treatment. The fresh weight (FW) of the leaves was weighed first. The leaves were then immersed in distilled water for 10 h to obtain the saturated fresh weight (TW). They were then dried in a 60 °C oven for 12 h until the leaves reached a constant weight, and the dry weight (DW) was measured [92]. Finally, the relative water content was calculated using the following formula:

RWC = (FW − DW)/(TW − DW) × 100%(1)

The relative electrolyte leakage (REL) was measured using an EC215 conductivity meter (Markson Science, Inc., Del Mar, CA, USA) [59]. The leaves were immersed in deionized water to determine their initial conductivity (L1) and then boiled before their final conductivity (L2) was determined, as shown below:

REL (%) = L1/L2 × 100%(2)

The content of MDA was determined using the thiobarbituric acid (TBA) method. A total of 0.1 g of fresh leaves was ground in liquid nitrogen, and 400 μL of 5% trichloroacetic acid (TCA) was added. The mixture was centrifuged at 6000 rpm at 4 °C for 15 min, and the supernatant was collected. A volume of 1 mL of 0.5% TBA was added. The solution was mixed thoroughly and incubated in a boiling water bath for 30 min. It was then cooled in an ice bath and centrifuged at 5000 rpm for 15 min. The absorbance was measured at 532 nm, 600 nm, and 450 nm using a UV1901PC ultraviolet–visible spectrophotometer (Shanghai Aoxi Scientific Instrument Co., Ltd., Shanghai, China) [93].

The content of free proline was determined using the sulfosalicylic acid method. A volume of 3 mL of 3% sulfosalicylic acid was added to 0.1 g of the leaves and extracted with boiling water for 10 min. The solution was then filtered. A volume of 2 mL of glacial acetic acid and 2 mL of 2.5% acidic indophenol solution was added to the filtrate. The solution was boiled for 30 min, cooled, and then extracted with 4 mL of toluene. It was then centrifuged at 3000 rpm for 5 min. The absorbance of the upper toluene layer was measured at 520 nm, and the content of proline was determined based on a proline standard curve [94].

The content of soluble sugars was determined using the anthrone method. A volume of 10 mL of distilled water was added to 0.1 g of the leaves, extracted with boiling water for 20 min, and diluted to 100 mL. A volume of 5 mL of 0.2% anthrone reagent was added to 1 mL of the extract. It was then incubated with boiling water for 10 min and cooled. The absorbance was measured at 625 nm, and the content of soluble sugars was calculated using a glucose standard curve [95].

The content of soluble protein was determined using the Coomassie Brilliant Blue method. A total of 0.1 g of leaves was sampled, ground in liquid nitrogen, and 5 mL of distilled water was added to the homogenate. The samples were centrifuged at 3000 rpm for 10 min. A volume of 5 mL of Coomassie Brilliant Blue G-250 was added to 1 mL of the supernatants. A volume of 5 mL of Coomassie Brilliant Blue G-250 was added to 1 mL of the supernatant. The mixture was incubated statically for 2 min, and the absorbance was measured at 593 nm. The content was calculated using a standard curve of bovine serum albumin [96].

The SOD, POD, and CAT were assayed by adding 0.1 g of the leaf tissue to pre-cooled potassium phosphate buffer (50 mmol/L, pH 7.0). The mixture was ground thoroughly in an ice bath and then centrifuged at 12,000 rpm at 4 °C for 20 min. The supernatant was considered to be the enzyme extract.

The CAT was assayed by UV absorption. A volume of 0.1 mL of the enzyme solution was added to 3 mL of the reaction mixture that contained 200 mL of 0.15 M PBS at pH 7.0 and 0.3092 mL of 30% hydrogen peroxide (H_2_O_2_). The change in absorbance at 240 nm over 60 s was measured using a control of the phosphate buffer [97].

The POD was assayed using the guaiacol method. A volume of 3 mL of reaction solution that contained 200 mL of 0.2 M PBS at pH 6.0, 0.076 mL of guaiacol (Beijing Coolaber Technology Co., Ltd., Beijing, China), and 0.112 mL of 30% H_2_O_2_ was added to 30 µL of enzyme solution. The change in absorbance at 470 nm was measured for 60 s with a blank of phosphate buffer [93].

The SOD was assayed using the nitroblue tetrazolium (NBT) reduction method. A volume of 3 mL of a reaction mixture that contained 162 mL of methionine, 0.6 mL of disodium EDTA, 5.4 mL of thiosulfate, 6 mL of NBT, and 6 mL of riboflavin was added to 30 µL of enzyme solution. The test tube was placed in a light box and treated with 4000 lux for 20 min. Two control tubes were simultaneously placed under the light. One contained 3 mL of reaction mixture and 30 µL PBS (without enzyme solution), which served as the maximum photoreduction control after illumination. The other tube only contained buffer and was kept in the dark as the zero-value control. After the reaction, the absorbance at 560 nm was measured and adjusted to zero using the unilluminated control tube [98].

### 4.7. Statistical Analysis

All the data were initially collated using Microsoft Excel 2021 (Microsoft, Redmond, WA, USA). The statistical analyses were performed using SPSS 18.0 (IBM Corporation, Armonk, NY, USA), and the figures were generated using GraphPad Prism 8.0 (GraphPad Software, San Diego, CA, USA). The data were expressed as the mean ± SD. A Duncan’s multiple range test was used to determine significant differences between the WT and transgenic lines. Significant differences between the WT and transgenic plants are indicated as follows: ns (*p* > 0.05), * *p* < 0.05. ** *p* < 0.01. *** *p* < 0.001. All the error bars in the figures represent SD.

## Data Availability

The original contributions presented in this study are included in the article and Supplementary Materials. Further inquiries can be directed to the corresponding author [lijin@shzu.edu.cn (J.L.)].

## Supplementary Materials

The following supporting information can be downloaded at: https://www.mdpi.com/article/10.3390/ijms27020688/s1.
