# Correction: Bao et al. Overexpression of the *Pyrus sinkiangensis LEA4* Gene Enhances the Tolerance of *Broussonetia papyrifera* to the Low Temperature During Overwintering. *Int. J. Mol. Sci.* 2026, *27*, 688

**DOI:** 10.3390/ijms27093964

**Published:** 2026-04-29

**Authors:** Xiaoxia Bao, Xueying Yang, Xue Wang, Hongliang Xin, Qianqin Li, Saisai Wang, Wenwen Xia, Jin Li

**Affiliations:** College of Life Sciences, Shihezi University, Shihezi 832000, China; v2023bxx@163.com (X.B.); 15299925708@163.com (X.Y.); 17899934396@163.com (X.W.); xhl18899533171@126.com (H.X.); liqianqin314@163.com (Q.L.); wssshzu@163.com (S.W.); xiashzu@126.com (W.X.)

The following reference [1] has been retracted and removed from the original publication [1]:

1. Liu, X.; Li, A.; Yang, X.; Luo, G.; Zhu, J. PsHB7/12 Gene Participated in the Overwintering Process of Pyrus sinkiangensis through Negative Feedback Regulation of ABA. *Plant Physiol. Biochem*. *PPB* **2025**, *220*, 109440.

The removal affects the first sentence of the Introduction Section, which previously read as follows: “Pyrus sinkiangensis, also known as Korla Fragrant Pear, is a distinctive fruit tree in the Xinjiang region in China.”

The reference citation [1] has been deleted from this sentence. With this correction, the order of the remaining references has been adjusted accordingly. No other changes were made to the text.

The authors state that the scientific conclusions are unaffected. The correction was approved by the Academic Editor. The original publication has also been updated.

## References

[1] Bao X., Yang X., Wang X., Xin H., Li Q., Wang S., Xia W., Li J. (2026). Overexpression of the *Pyrus sinkiangensis LEA4* Gene Enhances the Tolerance of *Broussonetia papyrifera* to the Low Temperature During Overwintering. Int. J. Mol. Sci..

