# Supplementary material for: Overexpression of the Pyrus sinkiangensis LEA4 Gene Enhances the Tolerance of Broussonetia papyrifera to the Low Temperature During Overwintering
Source: Int J Mol Sci. 2026 Jan 9;27(2):688. doi: 10.3390/ijms27020688 (PMC12840780; doi:10.3390/ijms27020688)
Supplement: Supplementary file 1 [file ijms-27-00688-s001.zip › ijms-4036706-supplementary-xml.pdf]

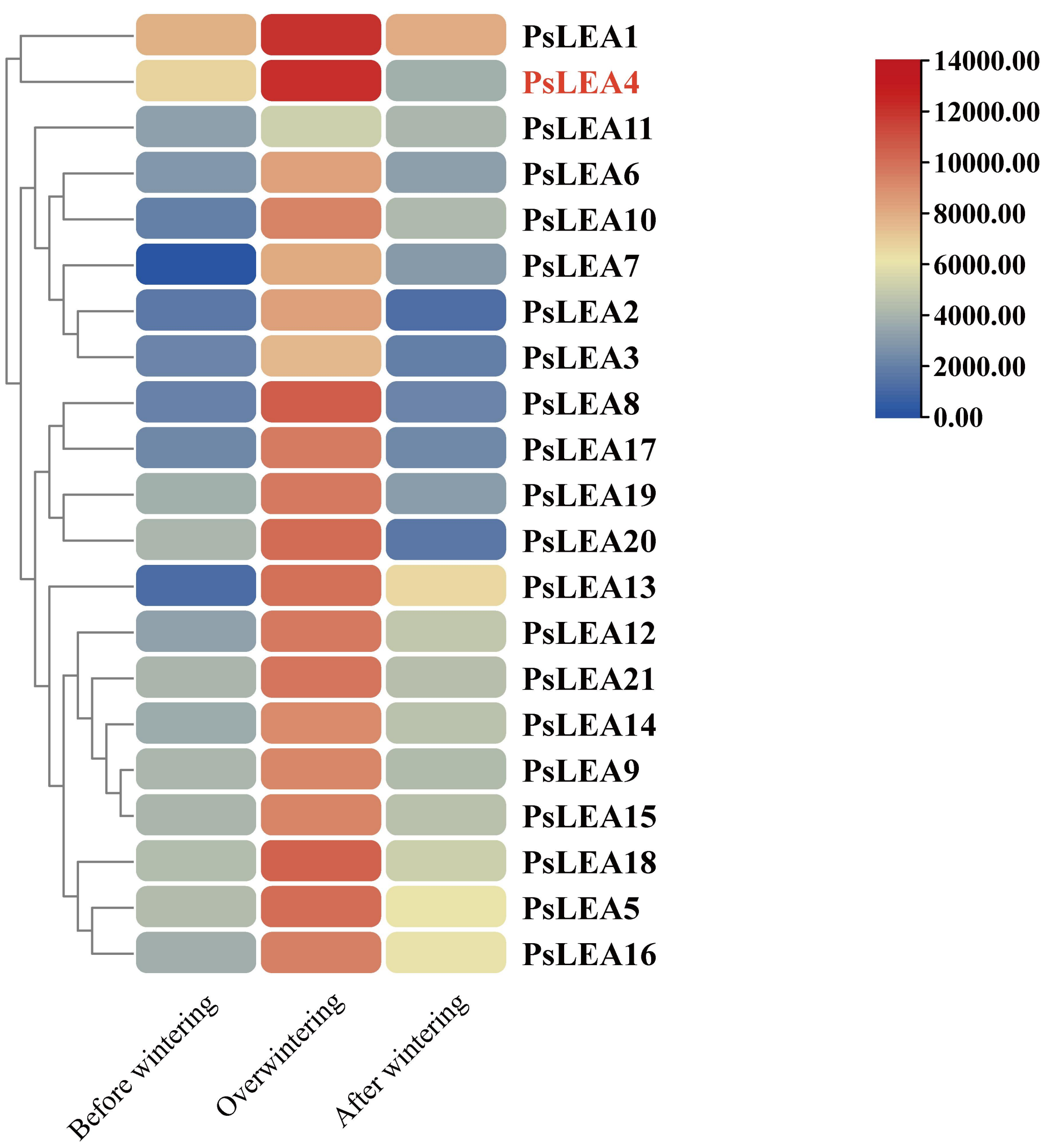

Figure S1. Expression Patterns of *PsLEA4* During the Overwintering Process.

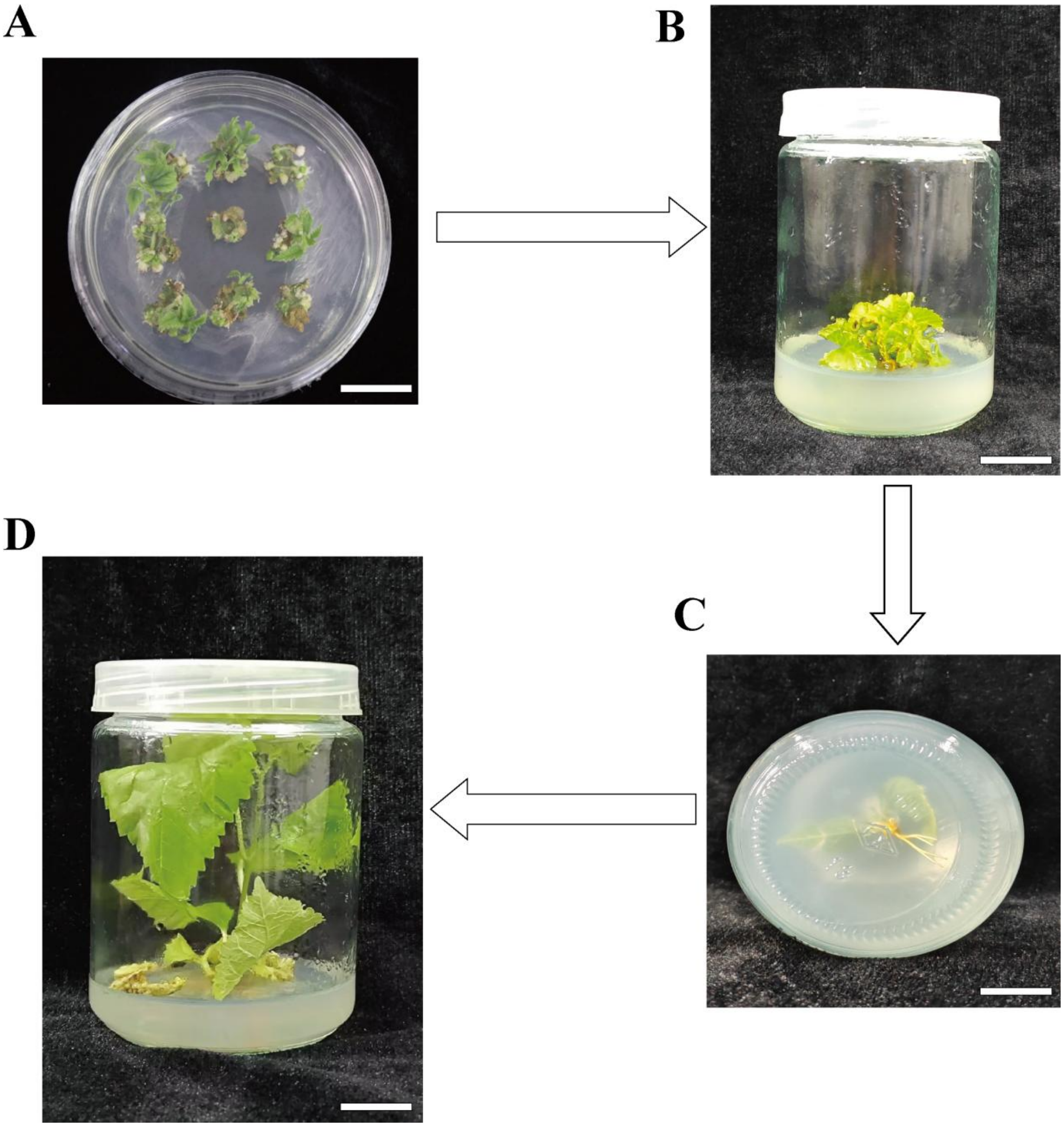

Figure S2. Genetic transformation process of PsLEA4-overexpressing paper mulberry. (A) Buds sprouting from callus tissue. (B) Inserting clustered buds into rooting medium. (C) PsLEA4-overexpressing paper mulberry developing root systems. (D) Plants ready for transfer to pots. Bar=5cm.

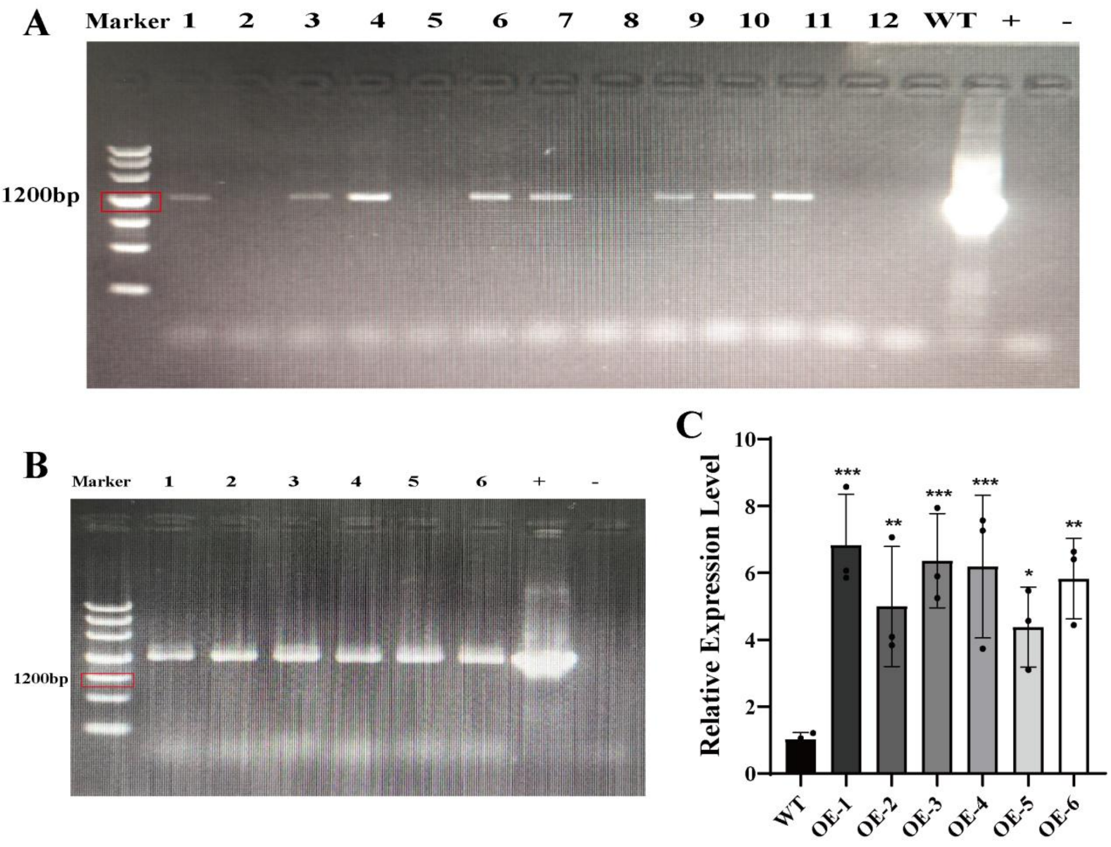

Figure S3. Identification of transgenic lines. (A) DNA-Level Identification. (B) RNA-Level Identification. (C) Identification of Relative Expression Levels.
